# Global climate change and invariable photoperiods: A mismatch that jeopardizes animal fitness

**DOI:** 10.1002/ece3.5537

**Published:** 2019-08-16

**Authors:** William H. Walker, Olga Hecmarie Meléndez‐Fernández, Randy J. Nelson, Russel J. Reiter

**Affiliations:** ^1^ Department of Neuroscience West Virginia University Morgantown WV USA; ^2^ Rockefeller Neuroscience Institute West Virginia University Morgantown WV USA; ^3^ Department of Cellular and Structural Biology University of Texas Health Science Center San Antonio TX USA

**Keywords:** circadian rhythms, climate change, light at night, photoperiod, reproduction, survival

## Abstract

The Earth's surface temperature is rising, and precipitation patterns throughout the Earth are changing; the source of these shifts is likely anthropogenic in nature. Alterations in temperature and precipitation have obvious direct and indirect effects on both plants and animals. Notably, changes in temperature and precipitation alone can have both advantageous and detrimental consequences depending on the species. Typically, production of offspring is timed to coincide with optimal food availability; thus, individuals of many species display annual rhythms of reproductive function. Because it requires substantial time to establish or re‐establish reproductive function, individuals cannot depend on the arrival of seasonal food availability to begin breeding; thus, mechanisms have evolved in many plants and animals to monitor and respond to day length in order to anticipate seasonal changes in the environment. Over evolutionary time, there has been precise fine‐tuning of critical photoperiod and onset/offset of seasonal adaptations. Climate change has provoked changes in the availability of insects and plants which shifts the timing of optimal reproduction. However, adaptations to the stable photoperiod may be insufficiently plastic to allow a shift in the seasonal timing of bird and mammal breeding. Coupled with the effects of light pollution which prevents these species from determining day length, climate change presents extreme evolutionary pressure that can result in severe deleterious consequences for individual species reproduction and survival. This review describes the effects of climate change on plants and animals, defines photoperiod and the physiological events it regulates, and addresses the consequences of global climate change and a stable photoperiod.

## 1 INTRODUCTION

Although not the first to describe humans' impact on the Earth's climate, Wallace Broecker is generally credited with coining the term “global warming” to describe the Earth's rising surface temperatures due to anthropogenic effects (Broecker, 1975). Prior to 1975, scientists favored the terms “climate change” or “climate modification” as it was unclear what effect human activities might have on the Earth's climate. Today, it is apparent that the Earth's mean surface temperatures are on the rise. Indeed, the Intergovernmental Panel on Climate Change (IPCC) reported a linear trend demonstrating a warming of 0.85°C in land and ocean surface temperatures from 1880 to 2012. This trend shows no signs of slowing as global surface temperatures at the end of the 21st century are predicted to rise as high as 4.8°C relative to 1850–1900 surface temperatures (IPCC, 2014).

The root of global climate change is likely anthropogenic in nature. Rise in global temperatures coincides with the Industrial Revolution and the emission of greenhouse gases, the most prominent of which is carbon dioxide (Maxwell, 1992). Undoubtedly, carbon dioxide emissions are increasing (Malik, Lan, & Lenzen, 2016); half of all the anthropogenic carbon dioxide emissions from 1750 to 2011 have occurred during the past 40 years, with the largest increases in greenhouse gas emissions from 2000 to 2010 (IPCC, 2014). Today, the US Environmental Protection Agency (EPA) estimates that carbon dioxide emissions from all human activities account for approximately 80% of the total annual greenhouse gas emissions within the United States. This accumulation of carbon dioxide in the Earth's atmosphere intensifies the greenhouse effect; thus, increased absorption and reflection of Earth's infrared energy emissions by a collection of greenhouse gases has led to increased surface temperatures (Anderson, Hawkins, & Jones, 2016).

Notably, the effects of global climate change are not uniformly distributed among latitudes, as higher latitudes are expected to warm at an increased rate relative to the global mean (IPCC, 2014). This is evidenced by the reductions in global glacier mass and the decline in size of Greenland and Antarctic ice sheets (Alley, Clark, Huybrechts, & Joughin, 2005; Oerlemans, 2005). Additionally, global climate change differentially affects seasons with a greater absolute increase in temperatures during the winter relative to the summer (Hughes, 2000). Indeed, the US National Oceanic and Atmospheric Administration (NOAA) records from 1970 to 2014 demonstrate a seasonal trend of winter temperatures warming twice the rate of the summer. Crucially, global climate change is not limited to increases in surface temperatures, as global climate change also encompasses alterations to the global hydrological cycle, and, consequently, global precipitation. Since 1950, precipitation has increased substantially in mid‐latitude land areas in the Northern Hemisphere. Analysis of satellite observations demonstrates a steady increase in overall precipitation and total atmospheric water, as a result of increased temperatures and the saturation of water vapor pressure (Liepert & Previdi, 2009; Wentz, Ricciardulli, Hilburn, & Mears, 2007). Furthermore, climate models predict a contrasting change in global precipitation; high latitudes, mid‐latitude wet regions, and the equatorial Pacific are expected to experience increased precipitation, whereas mid‐latitude and subtropical dry regions are expected to experience decreased precipitation (IPCC, 2014).

## 2 POTENTIALLY POSITIVE EFFECTS OF CLIMATE CHANGE ON PLANTS AND ANIMALS

On the surface, it may seem that global climate change has favorable effects on plants and animals. For plants, global climate change appears beneficial as it allows for the earlier onset of spring, prolonged growing season, as well as increased concentrations of atmospheric carbon dioxide, which is necessary for plant photosynthesis (Myneni, Keeling, Tucker, Asrar, & Nemani, 1997). Indeed, satellite data from a series of NOAA meteorological satellites have demonstrated, in Northern latitudes, increased plant growth during the summer and elevated plant respiration during the winter, which corresponded to rising temperatures and the lengthening of the active growing season (Myneni et al., 1997). Tree ring analyses in the Northern hemisphere have demonstrated increased tree growth during the past century (Briffa et al., 1998; Jacoby, D'Arrigo, & Davaajamts, 1996). Additionally, rising temperatures have allowed for the increased distribution and survival of some plant species; numerous studies have demonstrated an upward tree line migration and increased population growth at higher elevations and latitudes (Kullman, 2001; Lloyd, 2005; Mazepa, 2005; Meshinev, Apostolova, & Koleva, 2000; Smith, 1994; Sturm, Racine, & Tape, 2001; Walther et al., 2002). For example, Smith (1994) detailed the expansion of vascular plants, *Colobanthus quitensis* and *Deschampsia antarctica*, within Antarctica from 1960 until 1990. In Galindez Island, numbers of *D. antarctica* increased from 500 plants in 1964 to over 12,000 plants in 1990; similar effects were observed for *C. quitensis*. This expansion held true across multiple locations within Antarctica.

The favorable effects on plant growth due to global climate change can culminate in beneficial effects on animals. This is observed in tussocks cottongrass (*Eriophorum vaginatum*) and reindeer (*Rangifer tarandus*) inhabiting Seward Peninsula in Alaska (Cebrian, Kielland, & Finstad, 2008). Here, early snowmelt advanced the time of flowering of *E. vaginatum*, which serves as food for *R. tarandus*, and altered its nitrogen concentrations throughout inflorescence, effectively modifying its chemistry as evidenced by enhanced digestibility of the plant. Modeling suggests that an increase in digestibility of the plant will result in a twofold rise in dry matter intake, which will translate into an increase in reindeer weight, during the critical period of late winter to early spring, during which reindeer prepare for reproduction and breed (Cebrian et al., 2008). This increased body weight would support the recovery of fat and protein loss during the winter. Moreover, the replenishing of these nutrients would allow for a rise in milk production in females (Chan‐McLeod, White, & Holleman, 1994), resulting in positive cascading effects by increasing healthy calf growth, survival, and reproduction of the species.

Additional favorable effects have been observed on insects via earlier onset of insect flight periods, acceleration of development rates, enhanced winter survival, and expansion of range (Robinet & Roques, 2010). For example, more than 70% of butterfly species examined in the United Kingdom, Spain, and California have demonstrated an advancement in onset of flight (Forister & Shapiro, 2003; Roy & Sparks, 2000; Stefanescu, Peñuelas, & Filella, 2003). Similar advancements in first flight have been observed across other insect species including bees (*Apis mellifera*), fruit flies (*Bactrocera oleae*), and beetles (*Leptinotarsa decemlineata*; Gordo & Sanz, 2005).

Acceleration in development rates is expected, particularly among polyvoltine species (Robinet & Roques, 2010). For instance, from 1971 to 1996 the time required for the North American spruce beetle (*Dendroctonus rufipennis*) to reproduce has halved due to increased temperatures (Berg, Henry, Fastie, Volder, & Matsuoka, 2006). Additionally, enhanced winter survival has been reported in beetles (*D. rufipennis* and *Ips typographus)*, moths (*Thaumetopoea pityocampa*), aphids (*Myzus persicae*), and mosquitos (*Aedes albopictus*; Bale et al., 2002; Berg et al., 2006; Karuppaiah & Sujayanad, 2012; Robinet & Roques, 2010; Rochlin, Ninivaggi, Hutchinson, & Farajollahi, 2013). Remarkably, these changes coincide with the range expansion reported in dragonflies, grasshoppers, lacewings, butterflies, mosquitos, and moths (Hickling, Roy, Hill, Fox, & Thomas, 2006; Jepsen, Hagen, Ims, & Yoccoz, 2008; Menéndez, 2007; Rochlin et al., 2013).

## 3 DETRIMENTAL EFFECTS OF CLIMATE CHANGE ON PLANTS AND ANIMALS

Just as some of the effects of climate change might seemingly benefit some species, not all organisms and phenophases are able to respond to temperature and precipitation changes and adapt accordingly. For instance, despite climate change increasing the number of days above freezing and lengthening the growing season for plants, there are studies that suggest climate change may have detrimental effects on plant growth. Mora et al. (2015) used climate projection models to assess how rising temperatures in combination with water availability and solar radiation might affect suitable growing days for plants by the year 2100. The authors demonstrate that despite increasing the number of days above freezing by up to 7%, the number of suitable growing days would drop by up to 11%. Further, this reduction in suitable growing days was more profound in tropical areas, as the number of suitable growing days was projected to be reduced by up to 200 plant growing days per year, as warming will likely exceed the upper threshold for plant growth.

Additionally, rising temperatures are predicted to increase plant extinction, as plants can no longer track regions of suitable climate (Corlett & Westcott, 2013). Indeed, Corlett and Westcott (2013) propose that plant species will need to move greater than one kilometer per year to keep up with climate change; this pace can only be met in extremely rare instances where plants possess the combination of characteristics including long dispersal distances and short time to maturation. The harmful effects of global climate change on plants can also occur via indirect mechanisms. Harvell et al. (2002) suggest that rising temperatures and milder winters will have detrimental effects on plant species by increasing the winter survival of plant pathogens, accelerating their development, and expanding pathogen range. Indeed, studies examining the effects of climate change on plant diseases have concluded that rising temperatures likely increase plant disease severity. This is evidenced by *Phytophthora cinnamomic* causing more severe root rot at higher temperatures and a 14‐year field study in England demonstrating greater defoliation in warmer years by the Dutch elm disease fungus (*Ophiostoma novoulmi*; Brasier, 1996; Sutherland, Pearson, & Brasier, 1997).

Further indirect detrimental effects on plants include the expansion of invasive alien plant species (Dukes & Mooney, 1999; Hobbs & Mooney, 2005). Dukes and Mooney (1999) propose these invasive species possess particular traits which allow them to better capitalize on components of global climate change such as increases in CO_2_ concentrations and nitrogen deposition. Indeed, Smith et al. (2000) established that the presence of elevated carbon dioxide increased above‐ground production and seed rain in invasive annual grass compared to several species of native annuals. Furthermore, Nagel, Huxman, Griffin, and Smith (2004) demonstrate a stimulation of invasive grass species in the presence of carbon dioxide, with no response in cohabitating native species.

The adverse effects of global climate change are not limited to plants. One of the most worrisome consequences of climate change is the extinction of animal species. Although currently there is no meaningful evidence that climate change has led to species extinction, there is an abundance of evidence that demonstrates that climate change has led to population declines. This is particularly true for populations of animal species which are limited in range due to geographic constraints (i.e., high‐altitude mountainous species and Arctic/Antarctic species; Derocher, Lunn, & Stirling, 2004; Parmesan, 2006; Regehr, Lunn, Amstrup, & Stirling, 2007; Stirling, Lunn, & Iacozza, 1999; Trivelpiece et al., 2011; Wiig, Aars, & Born, 2008). For instance, Stirling et al. (1999) examined how the population ecology of polar bears (*Ursus maritimus*) in the Western Hudson Bay relates to climate change. The authors concluded that the physical condition and natality of polar bears had significantly declined from 1981 to 1998. This decline was associated with earlier sea ice breakup as a result of warming air temperatures.

Studies also have observed similar declines in populations of Antarctic Adélie, chinstrap, and emperor penguins (*Pygoscelis adeliae*,* Pygoscelis antarcticus*, and *Aptenodytes forsteri*, respectively; Barbosa, Benzal, León, & Moreno, 2012; Barbraud & Weimerskirch, 2001; Trivelpiece et al., 2011). Moreover, population declines have been reported in caribou (*Rangifer tarandus)*, reindeer (*Rangifer tarandus)*, Brünnich's guillemots (*Uria lomvia*), common eiders (*Somateria mollissima*), and arctic foxes (*Vulpes lagopus*) among others (Descamps, Strøm, & Steen, 2013; Killengreen et al., 2007; Merkel, 2004; Vors & Boyce, 2009). Additionally, climate change has been associated with declines in mountainous animal species such as pikas (*Ochotona princeps*) in the Western United States, Apollo butterflies (*Parnassius apollo*) in France, and harlequin toads (*Atelopus* sp.) and golden toads (*Bufo periglenes*) in the mountains of Costa Rica (Beever, Brussard, & Berger, 2003; Descimon, Bachelard, Boitier, & Pierrat, 2005; Pounds et al., 2006). These reductions in population size are likely due to a myriad of climate related factors including reductions in suitable habitat, changes in food sources and availability, alterations in predator–prey interactions, range expansion of competing species, and increase in pathogen survival and development (Davidson et al., 2011; Derocher et al., 2004; Dirnböck, Essl, & Rabitsch, 2011; Gilg, Sittler, & Hanski, 2009; Killengreen et al., 2007; Mallory & Boyce, 2017; Pounds et al., 2006; Stirling et al., 1999; Thomas et al., 2004; Tulp & Schekkerman, 2008; Wilson et al., 2005). For instance, a common hypothesis to explain the reductions in populations of Arctic foxes is the expansion of the range of the red fox (*V. vulpes*) to Arctic regions due to climate change. This range expansion of the larger sized red fox has had detrimental consequences on Arctic fox populations as they compete for similar food sources and den sites (Hersteinsson & Macdonald, 1992; Killengreen et al., 2007). Additionally, Pounds et al. (2006) concluded with very high confidence that in the mountainous regions of Costa Rica the expansion of the pathogenic fungus (*Batrachochytrium dendrobatidis)*, due to climate change, was responsible for the population decline and extinction of 67% of the 110 species of the harlequin toad. Furthermore, reductions in suitable habitat due to climate change have been proposed as a driving factor in the decline of diverse species such as polar bears and butterflies (Derocher et al., 2004; Parmesan, 2006; Wilson et al., 2005).

## 4 CLIMATE CHANGE AND PHOTOPERIOD

To our knowledge, prevailing models for predicting the impacts of climate change on organisms have yet to thoroughly integrate the interplay of factors other than temperature and precipitation that contribute to how organisms respond to the “new,” shorter and warmer winters. It is abundantly evident that climate has a profound effect on organisms, as it serves as a cue for life history events. Climate change has resulted in global variations in the onset of seasons, delaying winter and advancing spring, thereby, providing a wider range of favorable temperatures for some organisms, resulting in the expansion of their growing seasons, but creating a misalignment between species and their ecological interactors (Donnelly, Caffarra, & O'Neill, 2011). Although nutrition, water, and temperature are the ultimate drivers of seasonal rhythms, plants and animals have evolved remarkably similar mechanisms to use day length (photoperiod) as a proximate cue for predicting the occurrence of these ultimate factors. Although a crucial cue influencing organisms’ phenophases and life histories, photoperiod has not been examined thoroughly in relation to climate change. This review will primarily focus on photoperiod‐induced annual events in birds and mammals.

Photoperiod refers to day length, which varies with latitude and seasons. It is governed by Earth's rotation around the Sun and its tilt. With this continual rotation, the hemispheres receive differing exposure to sunlight, hence creating the different seasons characterized by varying day length and temperature. Day length does not perceptibly vary across years. Hence, it is a reliable cue for animals and plants to drive their nutritional, metabolic, and reproductive behaviors, which ultimately results in regular seasonal rhythms. Although photoperiod has no direct effects on fitness, it allows prediction of environmental factors that do directly affect fitness. Over evolutionary time, there has been selection for exquisite precision in photoperiodic regulation of physiology and behavior tied to environmental conditions (Stevenson, Prendergast, & Nelson, 2017). These photoperiod‐mediated patterns occur over various months every year in direct correlation with day length (Bartness & Wade, 1984; Bronson, 2009; Chemineau et al., 2008). Organisms under photoperiod‐driven rhythms depend on photic cues as driving factors for behavioral and physiological events. In mammals, light enters the eyes and stimulates the retinas, activating intrinsically photosensitive retinal ganglion cells (ipRGCs) that contain the photopigment, melanopsin, which responds especially to blue wavelength light. Despite their receptiveness to wavelengths of light, ipRGCs do not contribute to visual responses (Gooley, Lu, Chou, Scammell, & Saper, 2001; Hattar, Liao, Takao, Berson, & Yau, 2002). This information is relayed to the master circadian clock in the hypothalamus, the suprachiasmatic nucleus (SCN), via the monosynaptic retinohypothalamic tract (RHT; Moore & Lenn, 1972; Moore, Speh, & Card, 1995; Ralph, Foster, Davis, & Menaker, 1990; Sadun, Schaechter, & Smith, 1984; Stephan & Zucker, 1972b, 1972a). Subsequently, the SCN communicates with secondary oscillators in the brain, such as the pineal gland, pituitary gland, and other brain regions, which in turn, relay the photoperiodic signal to the rest of the body modulating sleep, endocrine responses (Moore & Eichler, 1972; Moore & Lenn, 1972), patterns of daily locomotor activity (Stephan & Zucker, 1972b, 1972a), and core body temperature (Scheer, Pirovano, Someren, & Buijs, 2005) among others. Hence, it follows that circadian rhythms can be tempered by altering biological light conditions. Importantly, having entrained circadian rhythms is critical for endogenous assessment of day length.

The annual cycle of changing day length is commonly used as a signal of the approaching and waning seasons. In many vertebrates, photoperiodic information is encoded by the central circadian clock located in the SCN (Hastings and Herzog, 2004). The SCN regulates the synthesis and release of melatonin from the pineal gland during the night; thus, day length differences are assessed by monitoring night length, which is encoded by the duration of melatonin secretion into the blood and cerebrospinal fluid (Pevet and Challet, 2011; Reiter, Tan, Kim, & Cruz, 2014). Relatively long durations of secreted melatonin encode long nights (or short days), whereas relatively short elevations of secreted melatonin encode short nights (or long days). Melatonin targets several brain regions to affect the phase of peripheral circadian clocks, as well as the central clock in the SCN, by altering expression of circadian clock genes (Pevet and Challet, 2011). Among the target sites of melatonin, the pars tuberalis (PT) of the pituitary stalk plays a key role in the photoperiodic pathway governing seasonal reproduction (Dardente, 2012). In mammals, a long‐day signal rapidly induces the strong peak of the transcription factor *Eyes absent 3 (Eya3*) in the PT (Dardente et al., 2010; Masumoto et al., 2010). EYA3 contributes to thyroid stimulating hormone (TSH) synthesis in the PT by activating transcription of *TSH b subunit* (*TSHb*). TSH acts on TSH receptor (TSHR)‐expressing cells in the basal hypothalamus to increase thyroid hormone (T_3_) availability. T_3_ interacts with hypothalamic peptides which eventually control the release of gonadotropins from the adenohypophysis leading to seasonal changes in gonadal size and function, as well as adjustments in body mass (Barrett et al., 2007). Although environmental factors such as temperature vary from year to year, annual changes in day length follow a predictable and consistent pattern. However, recent urbanization activity by humans has increased the prevalence of artificial light at night, rapidly changing the natural environment to which organisms must adjust (Hölker et al., 2010). Again, the role of day length in coordinating seasonal phenotypic changes has evolved with precision so that seasonal reproductive function coincides with optimal conditions for offspring and parental survival (Nelson, 1987). Exposure to light at night alters the daily melatonin cycle and other aspects of the circadian system and affects many photoperiod‐regulated physiological and behavioral responses (Navara and Nelson, 2007). Both synchronization of circadian clocks and photoperiodic time measurement depend on a distinct demarcation between light and dark. Modern illuminated skies prevent this demarcation (Dominoni, Borniger, & Nelson, 2016; Dominoni & Nelson, 2018).

## 5 EVENTS CUED BY PHOTOPERIOD

Studies on animals have revealed how photoperiod influences key events in their life history. For example, changes in day length affect metabolism causing fluctuations in body weight and related hormones. For nocturnal Syrian hamsters (*Mesocricetus auratus*), short days (10:14) promote body weight gain, as well as white and brown adipose tissue increase, independent of pinealectomy (Bartness & Wade, 1985). These findings suggest that melatonin is not required for this weight gain to occur. Djungarian hamsters (*Phodopus sungorus*) also experience photoperiod‐associated weight changes. They lose 30% of body weight with a 20% decrease in food intake over winter (Steinlechner, Heldmaier, & Becker, 1983); however, this decrease is not detrimental, but adaptive. Their size reduction allows the species to maximize its feeding efficiency by increasing the relative food requirements without increasing its absolute food intake. These cases are examples of seemingly opposite adaptive mechanisms, mediated by photoperiodic signals, to prepare these rodents for the winter months when food is scarce, and they need to effectively preserve energy.

Day length also serves as a cue for successful reproduction. Optimal synchrony of tissue‐specific peripheral circadian clocks, which may be impacted by perturbed photoperiodic cycles, is also essential for successful reproduction, as food and nutrient availability is key to fitness and survival (Reiter, Tan, Korkmaz, & Rosales‐Corral, 2013); hence, birth and rearing must be aligned to the time when these resources are accessible, while conserving energy when they are not. Under natural winter photoperiod and temperature conditions, male Syrian hamsters (*Mesocricetus auratus*) experience gonadal regression mediated by the pineal gland (Reiter, 1973), through its release of melatonin (Reiter, 1991) and its downstream cascade.

During the past 30 years, evidence has mounted that artificial light during the night (ALAN) disrupts circadian rhythms and photoperiodic responses. Of note, ALAN does not alter the length of day, but does alter perceived day length. Abundant data demonstrate harmful effects caused by altering the natural photoperiod. For example, dim light at night (dLAN) alters short‐day regulation of reproduction in male Siberian hamsters (*P. sungorus*; Ikeno, Weil, & Nelson, 2014); specifically, dLAN blunted nocturnal activity and altered the expression of genes implicated in photoperiodic response including, *Mel‐1a* melatonin receptor, *Eyes absent 3*, *thyroid stimulating hormone receptor*, *gonadotropin‐releasing hormone*, and *gonadotropin‐inhibitory hormone*. Additionally, these changes were associated with shifts in circadian clock gene expression (*Period1*), and alterations in gonadal mass, sperm numbers, pelage color, and pelage density (Ikeno et al., 2014). Similar changes in reproduction and mating have been demonstrated in *Drosophila melanogaster* (McLay, Nagarajan‐Radha, Green, & Jones, 2018), dLAN prolonged courting behavior, and altered oviposition patterns. LAN exposure of great tits (*Parus major*) and European blackbirds (*Turdus merula)* advanced the timing of vernal gonad growth (Dominoni et al., 2018; Dominoni, Quetting, & Partecke, 2013).

The effects of disrupted photoperiod due to LAN are not limited to reproduction. Numerous studies have demonstrated changes in behavior and immune function, which in the wild would likely lead to reduced fitness. For example, mice housed in dim light at night consume food at the “wrong” time of day (during the day), as well as reduce their avoidance of open field conditions, two behaviors which are maladaptive for small nocturnal prey species (Fonken et al., 2009, 2010; Nelson & Chbeir, 2018). Further, LAN exposure has detrimental effects on both the innate and adaptive immune systems. Japanese quail (*coturnix japonica*) housed in constant light demonstrated suppressed cell‐mediated immune response and humoral immune response when challenged with an antigen (Moore & Siopes, 2000). Similar deficits in immune function have been demonstrated in cockerels maintained in constant light and rats exposed to LAN. Specifically, LAN exposure suppressed antibody production in cockerels and reduced cytotoxic activity of natural killer cells in rats (Kirby & Froman, 1991; Oishi et al., 2006). Notably, given the pervasive nature of LAN in today's society, there will likely arise synergistic detrimental effects on individuals due to global climate change and exposure to light at night.

## 6 EFFECTS OF CLIMATE CHANGE WITH A STABLE PHOTOPERIOD

Despite the impact on temperature and precipitation, global climate change has no effect on day length, a key *Zeitgeber* (time giver) used by both plants and animals to time seasonal transitions and growing periods (Andrews & Belknap, 1993). Hence, the mismatch between temperature and day length cues creates an additional confounding dimension for organismal development, reproduction, and survival. This leads to potential detrimental effects on individuals as seasonality and annual events are modulated by both temperature and photoperiod; therefore, the growing disparity between temperature and photoperiod misaligns this synchrony that defines seasons and cyclic events. Examples of these are as follows: reproduction not aligned with nutrient availability (Visser & Both, 2005; Visser, Noordwijk, Tinbergen, & Lessells, 1998), disrupted prey–predator and plant–pollinator interactions, and other symbiotic relationships (Donnelly et al., 2011; Van Asch & Visser, 2006; Visser & Both, 2005).

Desynchronization of offspring birth and optimal nutrient availability has been reported in great tits (*Parus major*) in the Netherlands (Visser et al., 1998). Despite consuming a variety of insects as part of their diet, great tits preferentially feed their young protein‐rich caterpillars. Driven by photoperiod cues during the spring, the great tit lays its eggs at a time that provides an adequate interval before hatching; this aligns with the timeframe during which caterpillar biomass availability is at its maximum. However, during the period of 1973–1995 an increase in temperature during the post‐egg‐laying period advanced the development and availability of caterpillars, without advancing hatch time of the great tits (Visser et al., 1998). Consequently, a mismatch between offspring nutritional requirements and their availability ensued. It follows that great tits are now confronted with an evolutionary pressure to alter its reproductive phase to align with food availability. Clearly, to ensure the optimal survival of their offspring, the great tit is forced to advance its time of egg laying to match abiotic changes that cause the premature availability peak of its preferred fare. The laying date is not the only determinate in hatching date; synchrony can potentially be accomplished by reducing clutch size, by reducing the interval between laying of the last egg and incubation onset, or by reducing the overall time of incubation (Visser et al., 1998).

Additional examples of asynchrony between offspring birth and optimal nutrient availability have been reported in blue tits (*Parus caeruleus*) populations in France (Thomas, Blondel, Perret, Lambrechts, & Speakman, 2001). As in great tits, the blue tits use photoperiodic cues to commence breeding and reproduction and preferentially feed their young protein‐rich caterpillars. Global climate change has led to an advancement in caterpillar development without a concurrent advancement in hatching of the blue tit; thus, this mismatch has increased the metabolic cost of rearing young tits beyond the sustainable adult limit. Coincidentally, and possibly causally related, there has been a drastic reduction in the number of adults in the breeding population.

Yet another example of how the increasingly warming climate has affected animals is the Greenland caribou (*Rangifer tarandus*). Caribou are seasonal migratory animals which use day length as a cue for migration to ranges that favor calf rearing due to peak readiness of trophic resources. However, plant growth is cued by temperature, and as a result of warming over the years, now occurs in advance of caribou migration; thus, as in previous examples, creating a misalignment between offspring production, rearing, and peak resource availability (Post & Forchhammer, 2008). The result of this disparity has been an overall reduction in progeny production with an increase in progeny mortality. A similar mismatch between migration and food source availability due to climate change has been observed in the pied flycatchers of Western Europe (Both, 2010). Outside of breeding season, pied flycatchers (*Ficedula hypoleuca*) spend the year in Africa. Pied flycatchers use photoperiodic cues to prepare for and commence migration (Gwinner, 1996), thus trying to predict the initiation of spring at their breeding grounds in Western Europe. However, climate change has led to an advancement in the development and subsequent availability of caterpillars without a concurrent advancement in pied flycatchers' migration from Africa. This culminates in pied flycatcher chicks being fed a more varied and less nutrient‐rich diet, slowing their growth and reducing the number of birds that survive and return as breeders.

Further examples of climate‐induced asynchrony between seasons and circannual events include alterations in predator–prey interactions. Specifically, a recent decline in snowshoe hare (*Lepus americanus*) populations has been attributed to coat color mismatch (Mills et al., 2013; Pedersen, Odden, & Pedersen, 2017). Snowshoe hares use photoperiod cues to regulate and initiate molting via melatonin and prolactin signaling (Zimova et al., 2018). However, increasing temperatures, and thus reductions in snow cover, has resulted in a disparity between snowshoe hare coat color and dark snowless habitats. Unfortunately, the snowshoe hare has limited plasticity in molting time, thus leading to a 7% decline in survival rates due to increased visibility to predators and culminating in population declines (Zimova et al., 2018; Zimova, Mills, Lukacs, & Mitchell, 2014; Zimova, Mills, & Nowak, 2016). Similar detrimental disparities between coat color and the environment due to climate change have been reported in the Alpine rock ptarmigan (Lagopus muta), the Arctic fox (*Vulpes lagopus*), and snow leopards (*Panthera uncia*) and are presumed to occur in other animals such as lemmings (*Dicrostonyx*), weasels (*Mustela)*, and Arctic wolf (*Canis lupus*; Beltran, Burns, & Breed, 2018; Imperio, Bionda, Viterbi, & Provenzale, 2013; Zimova et al., 2018). Further examples encompassing disruption to aquatic animal phenophases, insect–plant interactions, and other cross‐kingdom interfaces have been elegantly reviewed in Donnelly et al. (2011).

Notably, most studies have examined the effects of climate change and a stable photoperiod on species inhabiting temperate and polar regions. Few studies have examined the effects of climate change, particularly alterations in photoperiod and precipitation, in subtropical and tropical species that time reproductive events during the rainy season (Bronson, 2009; Rissman, Nelson, Blank, & Bronson, 1987). Thus, future studies should examine the effects of climate change on reproduction and survival of subtropical and tropical species. Considering all things, it is undeniable that the ecological disruption left in the wake of global climate change can have a profound impact on species fitness and, ultimately, survival.

## 7 ADAPTIVE RESPONSES TO CLIMATE CHANGE AND A STABLE PHOTOPERIOD

As previously discussed, asynchrony between temperature‐defined seasons and circannual events as a result of global climate change can have a profound impact on species fitness and survival. Therefore, some species have developed adaptive responses to attempt to maintain synchrony. These adaptive responses include both phenotypic plasticity and heritable genetic changes. As previously discussed, great tits (*P. major*) have recently been confronted with a significant evolutionary pressure to alter its reproductive phase to align with food availability (Visser et al., 1998). Notably, great tits demonstrate individual plasticity in reproduction timing (Nussey et al., 2005). As a result, natural selection has occurred to favor highly plastic individuals by maintaining the greatest lifetime breeding success. Therefore, continued selection based on plasticity of egg‐laying time may alleviate this mismatch between reproductive timing and food availability.

Additional examples of adaptive responses to maintain synchrony between reproductive timing and food availability include Canadian red squirrels (*Tamiasciurus hudsonicus*) advancing their reproduction time to match with earlier spruce cone production, as well as multiple bird species in the United Kingdom advancing their time of egg laying to synchronize with earlier onset of spring (Crick et al., 1997; Réale, Berteaux, McAdam, & Boutin, 2003). Further adaptations in behavior have been reported that help to synchronize winter plumage mismatch in species of winter birds. For instance, rock ptarmigans (*L. muta*) in Canada dirty themselves when mismatched with snowless habitats, and Scandinavian willow ptarmigans (*Lagopus lagopus*) reportedly feed in areas that match their plumage, even when feeding grounds have inferior nutrients (Montgomerie, Lyon, & Holder, 2001; Steen, Erikstad, & Høidal, 1992). Notably, not all adaptations to maintain synchrony between temperatures, defined seasons and circannual events have occurred due to plastic phenotypes. Studies of pitcher‐plant mosquitos (*Wyeomyia smithii)* have demonstrated alterations in genetically controlled photoperiodic cues to enter winter diapause (Bradshaw & Holzapfel, 2001). Specifically, over the past 30 years northern populations of the pitcher‐plant mosquitos have evolved a shorter critical photoperiod to initiate winter diapause. Additionally, studies have demonstrated genetic selection for reproductive photoresponsiveness in deer mice (*Peromyscus maniculatus*; Desjardins, Bronson, & Blank, 1986).

## 8 CONCLUSIONS

**Figure 1:**
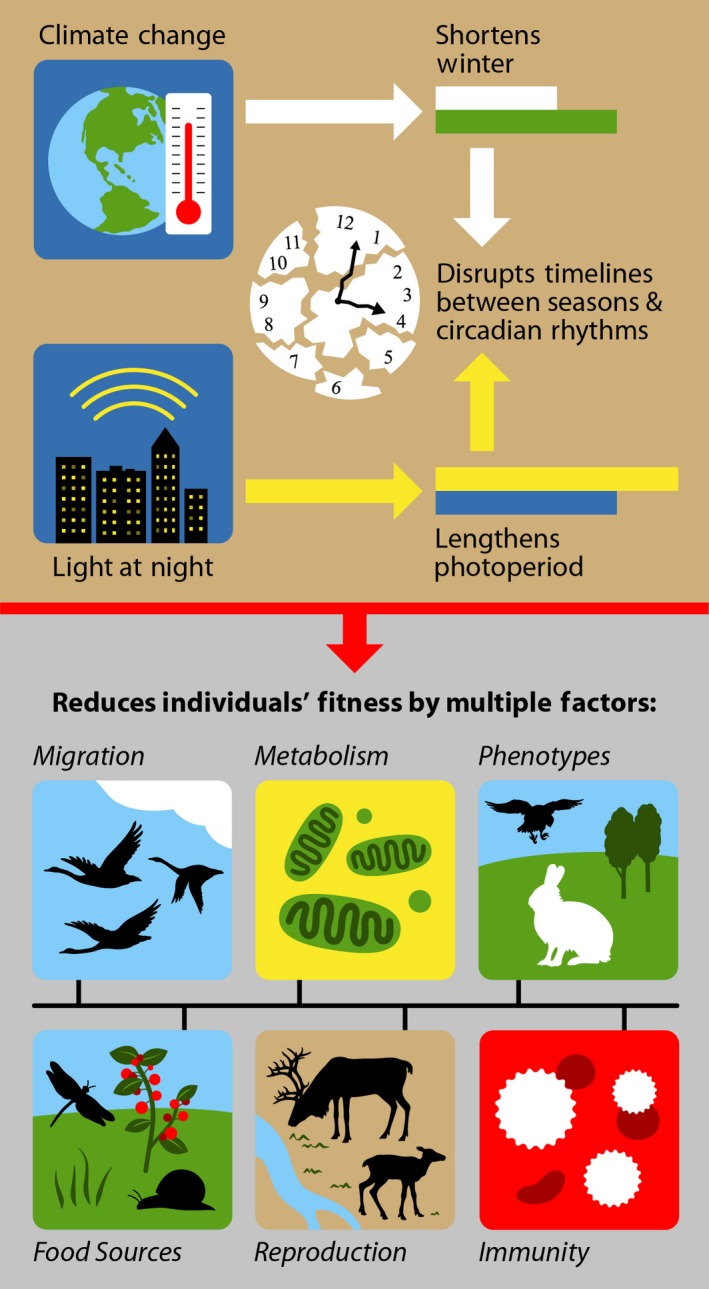
Climate change has provoked a mismatch between seasons and animals' seasonal rhythms; specifically, climate change has often phase‐shifted the growing season (e.g., shortened winter) without concurrent changes to photoperiod. Coupled with the effects of light pollution, which prevents individuals of photoperiod‐responsive species from determining day lengths, climate change presents extreme evolutionary pressure that can result in deleterious consequences. Specifically, climate change reduces individuals' fitness by altering predator–prey interactions, mistiming reproduction and migration with optimal nutrient availability, and in combination with light at night alters metabolic and immune function

It is indisputable that the Earth's surface temperature is rising and precipitation throughout the Earth is dynamic, and the cause of these shifts is likely anthropogenic in nature. Climate change has clear direct and indirect effects on both plants and animals, and it is possible that others exist which are yet unidentified or under examined. Of relevance, climate change in combination with a stable photoperiod presents extreme organismal evolutionary pressure and when organisms are unable to adapt or flexibly adjust, the result has severe deleterious consequences for individual species reproduction and, therefore, for species survival (Figure 1). Depending on the organism, its habitat, its ability to adapt, and its interactions with other species, the growing misalignment among temperature, precipitation, and photoperiod can translate into a falling out of synchrony between organisms and their native ecosystem. Moreover, not only are organisms under evolutionary pressures from abiotic factors in their environment (i.e., temperature and precipitation changes), but they are also subjected to the effects that other organisms in their environments might have due to their response and ability to adapt to these environmental changes. The ability of individuals to adapt will depend on the plasticity of the mechanisms underlying photoperiodic time measurement. Lack of plasticity in these mechanisms will likely lead to local extinctions.

## CONFLICT OF INTERESTS

The authors declare no conflicts of interests.

## AUTHOR CONTRIBUTIONS

WHWII, HMF, RJN, and RJR conceptualized the review. WHWII lead the writing of the manuscript. HMF, RJN, and RJR wrote and edited the manuscript.

## Data Availability

There are no data associated with this article.
